# Regulation of STAT1 and STAT4 Expression by Growth Factor and Interferon Supplementation in Sjögren’s Syndrome Cell Culture Models

**DOI:** 10.3390/ijms25063166

**Published:** 2024-03-09

**Authors:** Jean-Luc C. Mougeot, Thomas E. Thornburg, Braxton D. Noll, Michael T. Brennan, Farah Bahrani Mougeot

**Affiliations:** 1 Translational Research Laboratories, Oral Medicine, Oral & Maxillofacial Surgery, Atrium Health-Carolinas Medical Center, Charlotte, NC 28203, USA; thomas.thornburg@atriumhealth.org (T.E.T.); braxton.n@icloud.com (B.D.N.); mike.brennan@atriumhealth.org (M.T.B.); 2 Department of Otolaryngology, Head & Neck Surgery, Wake Forest University School of Medicine, Winston-Salem, NC 27157, USA

**Keywords:** STAT4, STAT1, cytokine regulation, interferons, EGF

## Abstract

Our goal was to investigate the effects of epidermal growth factor (EGF) and interferons (IFNs) on signal transducer and activator of transcription STAT1 and STAT4 mRNA and active phosphorylated protein expression in Sjögren’s syndrome cell culture models. iSGECs (immortalized salivary gland epithelial cells) and A253 cells were treated with EGF, IFN-alpha, -beta, -gamma, or mitogen-activated protein kinase p38 alpha (p38-MAPK) inhibitor for 0–24–48–72 h. STAT1 and STAT4 mRNA expression was quantified by qRT-PCR. Untreated and treated cells were compared using the delta-delta-CT method based on glyceraldehyde-3-phosphate dehydrogenase (GAPDH) normalized relative fold changes. phospho-tyrosine-701-STAT1 and phospho-serine-721-STAT4 were detected by Western blot analysis. STAT4 mRNA expression decreased 48 h after EGF treatment in A253 cells, immortalized salivary gland epithelial cells iSGECs nSS2 (*sicca* patient origin), and iSGECs pSS1 (anti-SSA negative Sjögren’s Syndrome patient origin). EGF and p38-MAPK inhibitor decreased A253 STAT4 mRNA levels. EGF combined with IFN-gamma increased phospho-STAT4 and phospho-STAT1 after 72 h in all cell lines, suggesting additive effects for phospho-STAT4 and a major effect from IFN-gamma for phospho-STAT1. pSS1 and nSS2 cells responded differently to type I and type II interferons, confirming unique functional characteristics between iSGEC cell lines. EGF/Interferon related pathways might be targeted to regulate STAT1 and STAT4 expression in salivary gland epithelial cells. Further investigation is required learn how to better target the Janus kinases/signal transducer and activator of transcription proteins (JAK/STAT) pathway-mediated inflammatory response in Sjögren’s syndrome.

## 1. Introduction

Sjögren’s syndrome (SS) is a chronic autoimmune disease impacting over 200,000 people in united states annually, targeting mucous membranes and glands of the eyes and mouth which can lead to other problems, such as dental cavities, oral thrush, and vision problems [1]. Salivary glands affected by SS commonly exhibit an epithelitis implying independent regulation of the immune response beyond reactivity to immune cell infiltration [2]. Epithelitis is marked by increased cytokine expression, limited Fas cell surface death receptor-mediated apoptosis, and p38-MAPK pathway activation promoting cluster of differentiation 40-mediated epithelial cell death [3,4,5]. In previous studies, the p38-MAPK pathway was demonstrated to be an upstream activator for phosphorylation and full transcriptional activity of STAT4 [6]. 

Determining the interactions between these pathways may allow for improved drug intervention treatment for SS patients. STAT4, involved in the JAK/STAT signaling pathway, is a confirmed SS susceptibility gene with active roles in cytokine expression and secretion, such as interferons (IFNs) and tumor necrosis factor-*alpha* in peripheral blood mononuclear cells (PBMCs) [7,8,9,10]. It is, however, unclear, the extent to which STAT4 and associated polymorphisms relate to immune cells and/or salivary gland epithelial cells during SS development. 

The JAK/STAT pathway mediates a variety of immune responses in both epithelial and immune related cell types [9]. Among the STAT proteins, there is little information describing how STAT4 specifically interacts with other STAT proteins within this pathway [11]. Moreover, this family of proteins interacts after phosphorylation, in which the proteins often dimerize and translocate to the nucleus where they regulate gene expression [12]. The JAK/STAT pathway, involving phospho-STAT4 activity, plays an important role in autoimmune mediated pathways and may facilitate aberrant inflammatory responses [13]. Additionally, cytokines such as IFNs bind and activate JAK-1/JAK-2, activating STAT proteins through phosphorylation, possibly propagating aberrant inflammatory responses in disease [14]. Targeting these factors or downstream targets in an appropriate manner within the salivary gland epithelia might serve to reduce the local inflammation and infiltration. Inhibition of JAK-1 has been studied for its potential as a possible treatment for SS [3,15]. JAK-1 inhibition may suppress both IFN type I and type II activities which can lead to downregulation of factors that promote infiltration of immune cells in salivary glands of SS patients [15]. 

Based on cross talk between pathways and potential effects on EGF, Wnt, and transforming growth factor-*beta* signaling pathways, it is inferred that small molecule inhibitors targeting JAK/STAT4 pathway may tamper with salivary gland epithelial cell homeostasis [16]. Therefore, this pathway needs to be better targeted to resolve inflammation and to preserve salivary gland function in SS [16]. EGF has been associated with the severity of intraoral manifestations in SS [17]. In addition, abnormal neovascularization in SS was shown to involve vascular endothelial growth factor receptor 2 (VEGFR2)/nuclear factor-*kappa* B (NF-κB) pathway leading to exacerbation of autoimmunity [18,19]. Also, there is an increased risk of cerebrovascular events and myocardial infarction in SS patients [20]. Although EGF and VEGF levels in saliva are not specific to the disease, SS patients exhibit a dysregulation of response to growth factors in addition to interferons [21]. 

The relationship between STAT4 and STAT1 and the response to growth factors and interferons are unclear. Therefore, since modulation of STAT signaling may provide new therapeutic avenues in general, our objective was to understand how this dysregulation affects expression of STAT4 and STAT1 at the mRNA level and phosphorylated protein levels using iSGECs and A253 cells line [22]. Additionally, since p38-MAPK and JAK/STAT pathways can act in concert in inflammation in multiple disease models, we tested the effects of p38 inhibitor on STAT1 and STAT4 expression in these cell lines [6,23,24].

## 2. Results

**Figure 1:**
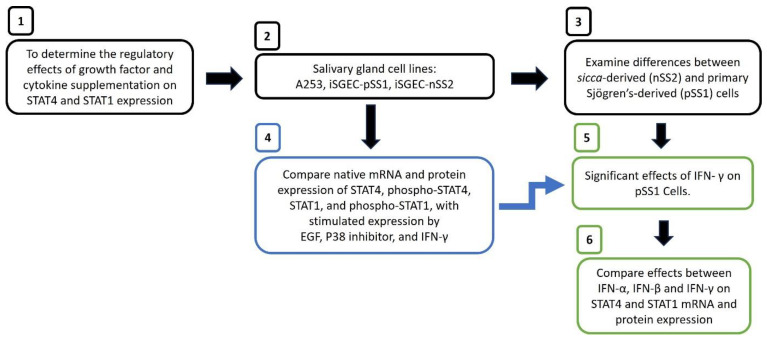
Determination of STAT1 and STAT4 expression and their phosphorylated forms in growth factors and interferon stimulated salivary gland cell line models for Sjögren’s Syndrome. Legend. (1) Experimental strategy used to determine regulatory effects of interferon proteins and growth factor supplementation on the expression of STAT4 and STAT1 is shown. (2) In this study, the A253 mucoepidermoid carcinoma of the salivary gland cell line was compared as a control to the iSGEC-pSS1 (primary Sjögren’s syndrome) and iSGEC-nSS2 (*sicca*) cell lines derived from primary salivary gland epithelial cells (SGECs) previously established in our laboratory. Previous studies have shown that interferons activate the JAK/STAT pathway resulting in the phosphorylation of STAT proteins and activating them as transcription factors. In Sjögren’s syndrome, STAT4 is known to contain susceptibility polymorphisms and to mediate cytokines (IFN, TNF-α) production. This led us to directly compare three cell lines: pSS1, an immortalized salivary gland cell line derived from a Sjögren’s patient (anti-SSA negative but focus score = 1.8); nSS2, an immortalized salivary gland cell line derived from a *sicca* patient (focus score = 0.16); A253, a mucoepidermoid salivary gland control cell line. (3) The pSS1 and nSS2 cell lines were directly compared to better understand their commonalities and differences (4) We investigated the effects of EGF, p38 inhibitor, and IFN-γ on STAT1 and STAT4 mRNA and active protein expressions, in all three cell lines. (5) Since the effects of IFN-γ on the pSS1 cell line were prominent for phospho-STAT1, we determined whether this effect was unique to IFN-γ, or if it was a more general response to interferons. (6) Protein and gene expressions following supplementation with IFN-α, IFN-β and IFN-γ were determined in each cell line.

The overall experimental design is presented in Figure 1. 

### 2.1. Regulation of STAT4 Expression by EGF and p38 Inhibitor

**Figure 2:**
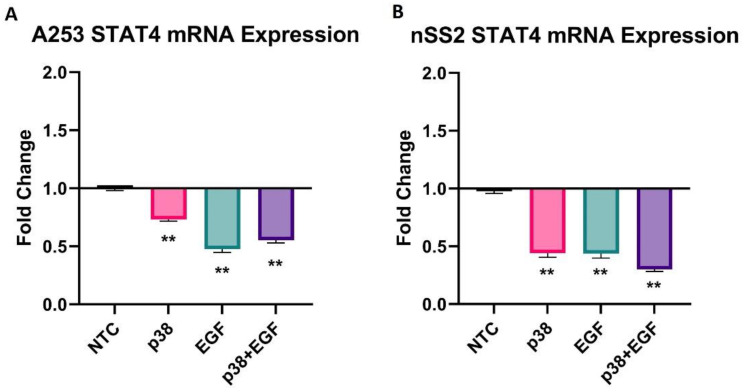
Effects of EGF and p38-inhibitor on STAT4 expression in salivary gland cell lines. Legend. (**A**) Expression of STAT4 mRNA following treatment of A253 cells with EGF and p38-I alone or in combination, is shown. (**B**) Results from supplementation with EGF and p38-I alone or in combination for nSS2 cells are shown. EFG is epidermal growth factor; p38-I is p38 inhibitor (SB203580); NTC is non-treated control. Error bars represent mean (+/−) standard error (SE) based on 3 independent replicates. Unpaired *t*-test was used to determine significance (** *p* < 0.01).

The mRNA expression of STAT4 was initially determined in A253 and nSS2 cells (Figure 2). The cells were cultured and treated with p38 inhibitor (20 µM) and EGF (10 ng/mL), and a combination of both. STAT4 expression was reduced in both cell lines in response to all three treatments of p38 inhibitor or EGF alone and the combination of both. In A253 cells the largest reduction resulted from EGF supplementation with an approximately two-fold reduction (Figure 2A). STAT4 expression in nSS2 ISGECs was similarly downregulated by EGF supplementation, but the combination of EGF and p38 inhibitor yielded an enhanced effect resulting in an approximately three-fold reduction (Figure 2B). The overall results indicate p38 inhibitor effects might be more pronounced on nSS2 cells.

### 2.2. Effects of EGF and IFN-γ on Protein Expression of Phospho-STAT4 and Phospho-STAT1

**Figure 3:**
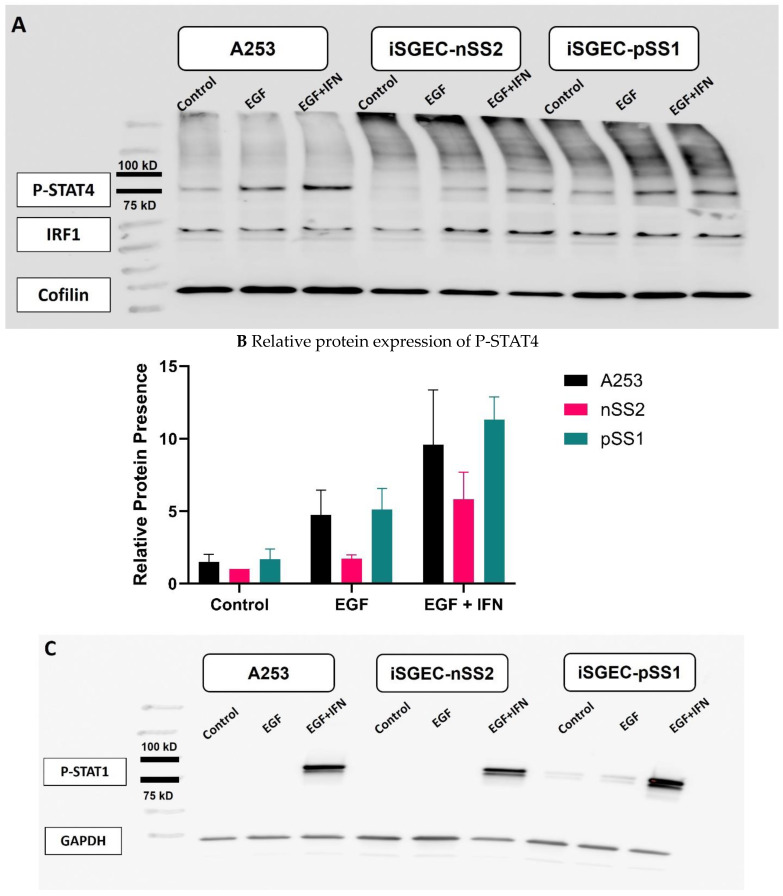

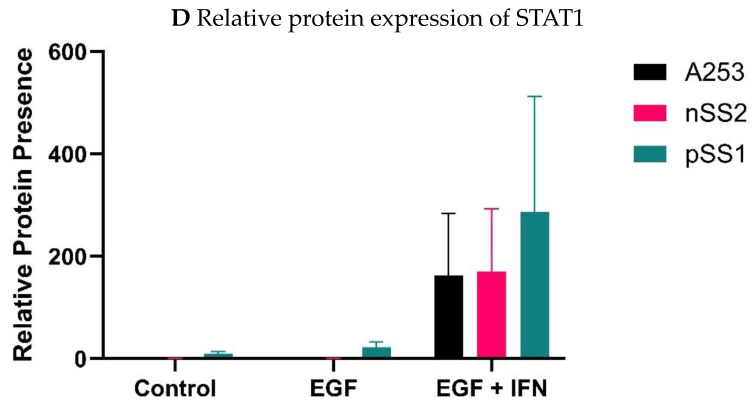
Western blot analysis of salivary gland cell lines treated with EGF and gamma-interferon. Legend. Representative Western blots and semi-quantitative Western blot analysis (from 3–4 independent experimental replicates) of cells treated with EGF and EGF combined with IFN-γ are shown. (**A**) Phospho-STAT4 (P-STAT4) and (**C**) Phospho-STAT1 (P-STAT1) protein levels were determined 72 h post-dosing with EGF and combined EGF and IFN-γ relative to untreated control, in A253, pSS1 and nSS2 whole cell lysates. (**B**,**D**) P-STAT4 and P-STAT1 presence was determined by semiquantitative analysis based on relative band intensity. Equal protein amounts were loaded in each lane and target bands were normalized to protein expression of cofilin or GAPDH. Loading controls and target proteins of the same blot were individually optimized for exposure requirements and reconstituted for imaging While incremental differences were observed for P-STAT4 across the three treatments, no changes were observed for interferon regulatory factor 1 (IRF1) (A). Effects from combined treatment with EGF and IGF were overall significant across the three cell lines compared to control and EGF treatment alone for both P-STAT1 and P-STAT4 (*p* < 0.01, Wilcoxon signed-rank test with Bonferroni correction).

To better understand the effects of EGF and IFN-γ supplementations in A253 and iSGECs on phospho-STAT4 and phospho-STAT1 protein production, the presence of both proteins was determined by Western blot. In all three cell lines, phospho-STAT4 presence was slightly increased by EGF (*p* = 0.11262)) and further increased by the combination of EGF and IFN-γ (*p* = 0.003906) (Figure 3A). Notably, A253 and pSS1 cells demonstrated an approximate average of 8- and 4-fold increase in phospho-STAT4 levels, respectively, after supplementation with both EGF and IFN-γ (Figure 3B). These effects are in the opposite direction compared to our described mRNA expression results for A253 and nSS2 cells in which STAT4 mRNA was downregulated in response to the addition of EGF (Figure 2). Phospho-STAT1 was expressed at lower levels in both A253 and nSS2 cells compared to pSS iSGECs (*p* < 0.05, paired *t*-test) (Figure 3C). All three cell lines displayed a large increase in phospho-STAT1 relative expression with supplementation of both EGF and IFN-γ combined, this effect being solely attributed to IFN-γ (Figure 3D). Of these, pSS1 iSGECs showed the largest presence of phospho-STAT1 compared to A253 cells and nSS2 iSGECs (Figure 3D).

### 2.3. Regulation of STAT1 and STAT4 mRNA and Active Protein Expression by Interferons α, β and γ

**Figure 4:**
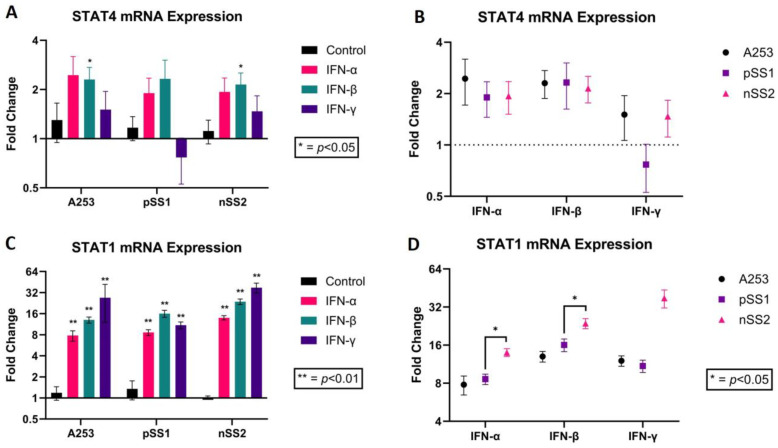
mRNA expression analysis of salivary gland cell lines A253, nSS2, and pSS1 treated with interferons α, β and γ. Legend. mRNA expression of STAT4 and STAT1 determined by RT-PCR was normalized to GAPDH. (**A**) Bar graph representing STAT4 mRNA relative expression in all cell lines treated with type I (IFN-alpha and -beta) and type II (IFN-gamma) interferons. (**B**) Plot showing differences in effects from type I and type II IFNs. (**C**,**D**) Bar graph representing STAT4 and STAT1 mRNA relative expression in all cell lines treated with type I (IFN-alpha and -beta) and type II (IFN-gamma) interferons and plot showing differences in effects of type I and type II IFNs. Error bars represent mean (+/−) standard error (SE) based on 9 independent experimental replicates. Mann–Whitney U-test was used to determine significance (** *p* < 0.01; * *p* < 0.05).

To further investigate the large effect of IFN-γ on STAT1 and STAT4 mRNA and protein expression, the effects of IFN-α and IFN-β protein supplementation were also assessed. The effects of IFN-α, IFN-β and IFN-γ on STAT4 and STAT1 mRNA expression in A253 cells, pSS1 and nSS2 ISGECs were determined. STAT4 expression was upregulated in all cell lines by IFN-α and IFN-β but was not changed by IFN-γ in pSS1 iSGECs (Figure 4A). 

While comparisons of STAT4 levels between cell lines did not show significant differences, there was a noticeable difference in expression between pSS1 cells and the other two cell lines when treated with IFN-γ (Figure 4B). In contrast, STAT1 expression was found to be upregulated in all three cell lines with supplementation of type I and type II IFNs (Figure 4C). There was a significant difference between pSS1 and nSS2 cells in STAT1 expression with type I IFN treatment (IFN-α and IFN-β), where nSS2 cells displayed higher expression (Figure 4D). This trend was also observed in the IFN-γ treatment group but was not found to be statistically significant. These data show that nSS2 cells respond to IFN proteins in a unique manner.

**Figure 5:**
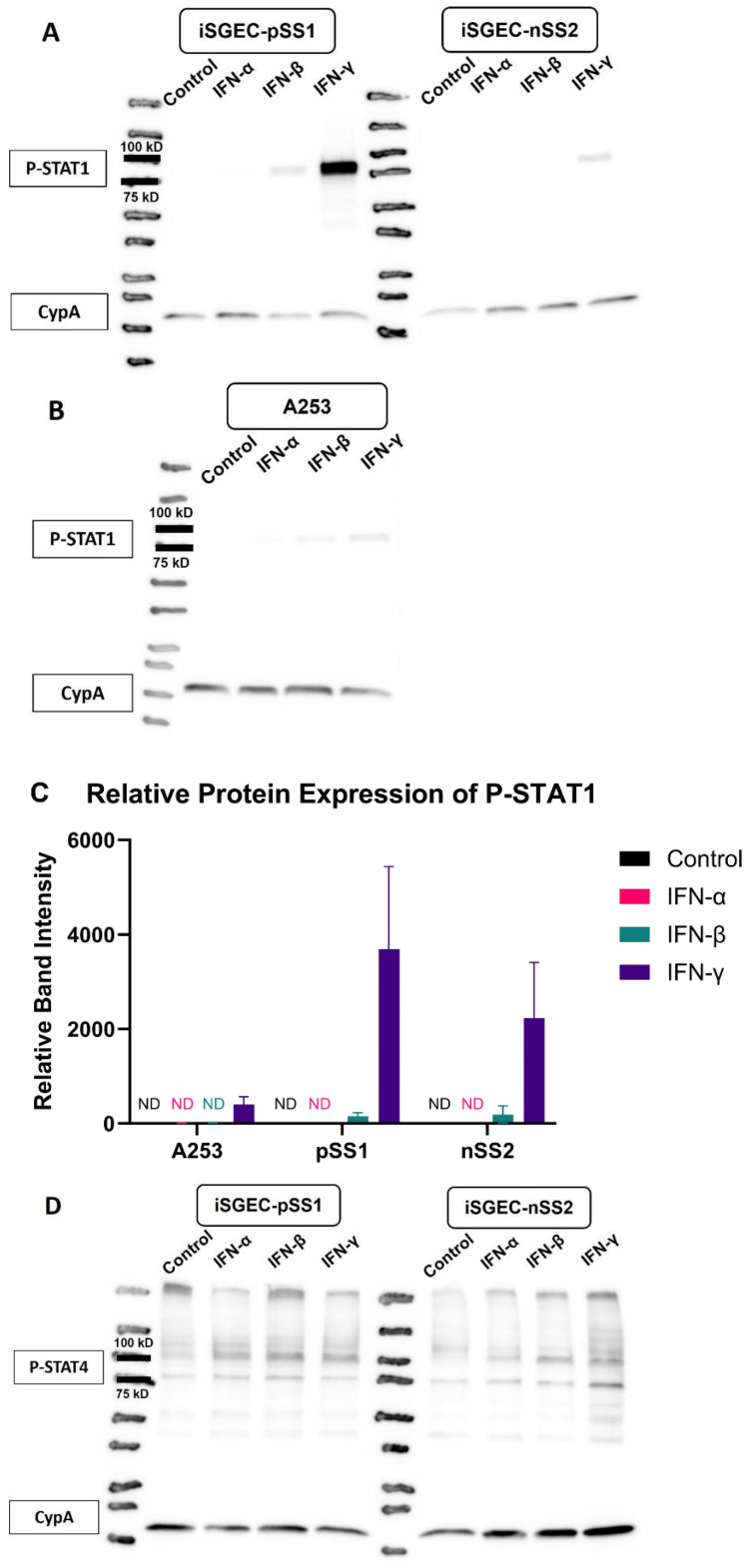

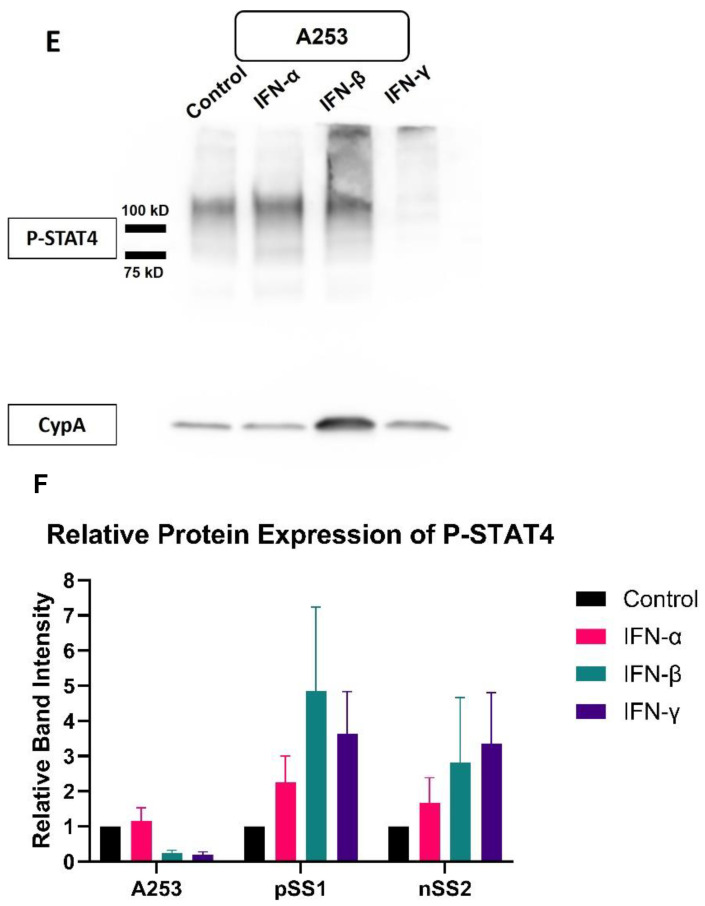
Active protein expression analysis of salivary gland cell lines treated with interferons α, β and γ. Legend. Representative Western blots and semi-quantitative Western blot analysis (from 3–4 independent experimental replicates) of IFN treated cells are shown. (**A**,**B**) Phospho-STAT1 protein levels were determined 72 h post dosing with IFN-α, IFN-β, and IFN-γ in A253, pSS1 and nSS2 whole cell lysates. (**C**) phospho-STAT1 (P-STAT1) presence was measured by semiquantitative analysis based on relative band intensity. (**D**,**E**) phospho-STAT4 (P-STAT4) protein levels were determined 72 h post dosing with IFN-α, IFN-β, and IFN-γ in A253, pSS1 and nSS2 whole cell lysates. (**F**) P-STAT4 expression was measured by semiquantitative analysis measuring relative band intensity. Equal protein amounts were loaded in each lane and target bands were normalized to protein expression of cyclophilin A. Error bars represent the mean (+/−) standard error (SE). However, the Control value was set to 1 by default without error bar for better representation of the differences between cell lines. Loading controls and target proteins of the same blot were individually optimized for exposure requirements and reconstituted for imaging. IFN-γ had a significant effect on P-STAT1 expression for pSS1 and nSS2 cell lines (*p* < 0.05, paired *t*-test with Bonferroni correction). There was a marginal effect of IFN-γ on P-STAT4 expression in pSS1 cells (*p* = 0.049). ND is not detected.

Following observation of an increase in STAT1 mRNA expression with type I and type II IFNs, expression changes of phospho-STAT1 and phospho-STAT4 were determined by Western blot analysis. Each cell line was grown with supplementation of IFN-α, IFN-β and IFN-γ and was harvested for protein after 72 h. Type I interferon IFN-β supplementation showed low relative expression in nSS2 cells and pSS1 cells for phospho-STAT1, whereas IFN-γ supplementation yielded a statistically significant increase in phospho-STAT1 in both pSS1 and nSS2 cells (Figure 5A). A253 cells were not significantly responsive to both type I and type II IFNs (*p* < 0.05). The relative expression of phospho-STAT1 based on the normalized control Cyclophilin A, is shown is Figure 5C. Western blots of phospho-STAT4 protein expression for the three cell lines are shown in Figure 5D,E. The relative protein expression for phospho-STAT4 is shown in Figure 5F. The data show a slight marginal effect of IFN-γ on pSS1 cells. 

## 3. Discussion

In this study, for the first time, we have shown differences in responses to EGF and interferons regarding the expression of STAT1 and STAT4 between *sicca* and SS patients’ derived iSGECs at both mRNA and active protein levels. Multiple genetic polymorphisms in the STAT1-STAT4 risk locus located on chromosome 2 have been confirmed for their association with Sjögren’s syndrome [25]. While both STAT1 and STAT4 have been associated with SS, it is unclear how polymorphisms affect these adjacent genes, whether in PBMCs or in salivary gland epithelial cells that are no longer considered as passive bystanders [26,27,28,29].

While we demonstrated differential reactivity to interferons and EGF between pSS1 and nSS2 iSGEC cell lines (more prominent for pSS1), we recognize that the SS-related biology of the cells will need to be confirmed in in vivo and ex vivo models for SS. In addition, Theander et al. 2015 [30] showed that SS autoantibodies may be present 20 years before SS diagnosis, which raises the question as to whether genetic polymorphisms, somatic mutations, or epigenetic changes may have occurred in these patients’ salivary glands that would promote autoimmunity. Ideally, single cell analysis would help the field to develop iSGECs that are representative of defined categories of *sicca* and SS patient categories, carrying recently confirmed/identified single nucleotide polymorphisms (SNPs) [25], or would have been engineered to carry such SNPs by clustered regularly interspaced short palindromic repeats (CRISPR) gene editing. Thus, an appropriate panel of iSGECs carrying SS-associated SNPs might be suitable for initial drug screening assays or assays which will contribute to the understanding of onset and progression of the disease. Moreover, the role of interferons in SS pathogenesis and clinical trials testing inhibitors of IFN-related pathways have been extensively reviewed by Del Papa et al. 2021 [31]. Whether polymorphisms impact immune cells or SGECs in patients, testing IFN reactivity of iSGECs from different sources might contribute to the understanding of inefficacy of drugs tested so far in clinical trials.

### 3.1. STAT4 mRNA Expression Downregulation by EGF and p38 Inhibitor

Previous studies in mice models for Sjögren’s syndrome have shown that treatment with p38 inhibitors have positive effects on the pathology of the disease [32,33,34]. In addition, there is an inverse correlation between EGF salivary levels and SS progression. Our results show downregulation of STAT4 at the mRNA level whether A253 or nSS2 cells were treated with EGF or p38 inhibitor alone or in combination, and to a greater extent for nSS2 cells. The significance of this result is unclear but might reflect an imbalance in JAK/STAT pathway [26]. Indeed, salivary gland epithelium homeostasis is maintained by numerous signaling pathways. EGF receptors are known to be involved in signaling pathways necessary for the TDL-4-mediated activation of downstream NF-κB pathway [26]. 

### 3.2. Effects of EGF and IFN-γ on Protein Expression of Phospho-STAT4 and Phospho-STAT1

The role of type I and type II interferons in the development of Sjögren’s syndrome may not be understated [31]. However, their effects on the production of active forms of STAT1 and STAT have been mainly investigated in PBMCs and infiltrating immune cells within the salivary gland [31]. In this study, EGF increased phospho-STAT4 in all three cell lines A253, nSS2, and pSS1, and to a larger extent when EGF was combined with IFN-γ, suggests an additive effect. Since, EGF was shown to reduce STAT4 at the mRNA levels (Figure 2), there is a possibility of regulation of the production of phosphorylated STAT4 protein. Such regulation might involve microRNA stabilization process or increased phosphorylation due to an unknown mechanism. It remains also unclear how the p38-MAPK pathway is tied to this mechanism. In addition, phospho-STAT1 was remarkably overexpressed in all three cell lines when EGF and IFN-γ were used in combination, although such effect was most likely due to IFN-γ. In this case, pSS1 showed the highest induction which was higher than in nSS2. The results suggest more prominent deregulation of the JAK/STAT pathway in pSS cells compared to nSS2 cells. This result, overall, warrants further investigation of the multi-protein transcriptional complex involved on chromosome 2 in absence or presence of polymorphisms. The effect of polymorphisms in STAT1 and STAT4 associated with Sjögren’s syndrome in cell culture models is currently under investigation in our laboratory. 

### 3.3. Regulation of STAT1 and STAT4 mRNA and Active Protein Expression by Interferons α, β and γ

We further investigated the interferon type I and type II responses in the three cell lines A253, nSS2, and pSS1 to determine whether there were significant differences in STAT1 and STAT4 mRNA expression. As shown in results, there were two noticeable differences. IFN-γ induced an opposite trend in STAT4 mRNAS expression in pSS1 cells compared to A253 and nSS2 cells, suggesting a fundamental difference in reactivity. Additionally, the results of 9 replicates showed variability in response to IFN-γ in pSS1 cells. Assuming minimal technical error, this result might reflect an intrinsic feature in pSS1 possibly associated with epigenetic changes upon cell passaging, as processing of outliers did not improve statistical significance. Furthermore, type I and type II interferons induced similar responses in all three cell lines regarding STAT1 mRNA expression. However, nSS2 and pSS1 cells behaved differently, with lower STAT1 mRNA expression in pSS1 compared to nSS2. The results suggest unique properties of pSS1 cells compared to nSS2, possibly reflecting Sjögren’s syndrome specific pathological features. 

At the active protein level, IFN-γ had the largest effect on the expression of phospho-STAT1 in pSS1 cells, confirming intrinsic differences between pSS1 and nSS2 cells. In contrast, there was a small effect by type I and II IFNs on the expression of phospho-STAT4, for which a trend of greater effect in pSS1 was observed. Overall, results show consistency among the differences observed at both mRNA and active protein expression levels for the two genes.

### 3.4. Limitations

This study used two immortalized cell lines derived from one sicca and one anti-SSA negative Sjögren patients’ salivary gland epithelial cells. Also, the study could have further benefitted from iSGECs derived from an anti-SSA positive Sjögren patient. Efforts in our lab are underway to circumvent these limitations. Interpretation of our data is in part limited due to insufficient data in animal models relevant to interferon and JAK/STAT related pathways in salivary gland epithelial cells. Additional experimentation using for instance siRNA and CRISPR knockdown technologies will be required to better understand the interaction between EGF, interferons, and the p38-MAPK pathway in regulating STAT1 and STAT4 and to characterize the impact of such interaction on JAK/STAT pathway in vitro and in vivo. 

## 4. Materials and Methods

### 4.1. Reagents

Reagents used included: Epilife media with HKGS (Human Keratinocyte Growth Serum; medium K) (ThermoFisher Scientific, Waltham, MA, USA), McCoys 5A Medium; medium 5A) (Cytiva, Marlborough, MA, USA), FBS (VWR International, Radnor, PA, USA), LB Agar with AMP, Human immortalized salivary gland epithelial cells (iSGECs) (established in our laboratory), salivary gland cell line A253 (ATCC—American Type Culture Collection, Manassas, VA, USA), EGF (ThermoFisher Scientific, MA, USA), p38 Inhibitor (SB203580) (Cell Signaling Technology, Danvers, MA, USA), Quick-RNA Miniprep Kit (Zymo Research, Irvine, CA, USA), HEX random primers (IDT—Integrated DNA Technologies, Coralville, IA, USA), DTT, SmartScribe reverse transcriptase kit (Takara Bio, San Jose, CA, USA), dNTPs (NEB—New England Biolabs, Ipswich, MA, USA), SYBR green master mix (Qiagen, Germantown, MD, USA), primer (IDT—Integrated DNA Technologies, IA, USA), Western blot transfer membrane (Cytiva, MA, USA), and antibodies (Supplementary Table S1). 

### 4.2. Cell Culture

Human iSGECs, and A253 (ATCC) were inoculated into media K and 5A (10% FBS), respectively, and treated with IFN-gamma (10 ng/mL) (Cell Signaling Technology), IFN-alpha (10 ng/mL) (Cell Signaling Technology), IFN-beta (10 ng/mL) (Proteintech, Rosemont, IL, USA), EGF (10 ng/mL) (ThermoFisher), and/or p38 inhibitor (20 µM) (Cell Signaling Technology) for 24 and 48 h. Cells were cultured in CO_2_ incubator (37 °C; 5% CO_2_). After 24 and 48 h incubation, the cells adhered to the well surface indicating successful recovery. Cells were then used for RNA extraction, conversion to cDNA for further qRT-PCR. 

### 4.3. Quantitative Real-Time RT-PCR

Total RNA was isolated from cells using the Quick-RNA Miniprep kit (Zymo) following the manufacturer protocol. RNA (500 ng) was reverse transcribed from each sample using SmartScribe reverse transcriptase kit (Takara) following the provided instructions. Random hexamers (IDT) and dNTP mix (NEB) were used. Expression levels of STAT4 and STAT1 were determined relative to GAPDH based on the ∆∆CT method using SYBR Green mix (Qiagen). qRT-PCR primers are listed in Supplementary Table S2. 

### 4.4. Western Blot 

Cells were grown in 6-well plates and were serum starved for 24 h. Treated and untreated cells were grown for 72 h before harvesting whole cell lysates for nuclear protein using MPER (Mammalian Protein Extraction Reagent) (ThermoFisher). Levels of target proteins, phospho-tyrosine-701-STAT1 and phospho-serine-721-STAT4 in whole cell lysates of treated and untreated iSGECs and A253 cells, were determined by Western blot. Primary antibodies were used at appropriate dilutions (Supplementary Table S1). Secondary antibodies anti-mouse IgG-HRP and anti-rabbit IgG-HRP were used according to primary antibodies. Supersignal West Pico and Femto solutions were used for signal detection (ThermoFisher). ImageQuant LAS4000 (GE) was used for imaging and Image Studio^TM^ Lite v2.5 (Li-COR Biosciences – U.S.) for data processing. 

### 4.5. Statistical Analysis

Differences of expression in the cell lines of qRT-PCR results relative to the control or between cell lines were analyzed by Mann–Whitney U-test (9 replicates for interferons treatments) or *t*-test (3 replicates for p38 and EGF treatments) (alpha = 0.05). Wilcoxon signed-rank test with Bonferroni correction was used on Western blot data normalized to the loading control to compare treatments with EGF alone and EGF and IFN-γ combined. Paired *t*-test with Bonferroni correction was used on Western blot data normalized to the loading control to compare treatments with IFN-α, IFN-β and IFN-γ relative to the experimental control.

## 5. Conclusions

Immortalized salivary gland epithelial cells derived from *sicca* and Sjögren syndrome patients may constitute useful tools to pinpoint and investigate specific disease mechanisms and genetic abnormalities. Indeed, *sicca* and Sjögren syndrome patients represent a broad spectrum of disease etiologies. Thus, future genomic single cell analysis may provide further clues as to how better target the JAK/STAT pathway to modulate the immune response to improve clinical outcomes in Sjögren’s syndrome patients. 

## Data Availability

The data presented in this study are available on request from the corresponding author. The data are not publicly available as no genome-wide data have been generated/analysed in this study.

## Supplementary Materials

The following supporting information can be downloaded at: https://www.mdpi.com/article/10.3390/ijms25063166/s1.
