# Supplementary material for: Regulation of STAT1 and STAT4 Expression by Growth Factor and Interferon Supplementation in Sjögren’s Syndrome Cell Culture Models
Source: Int J Mol Sci. 2024 Mar 9;25(6):3166. doi: 10.3390/ijms25063166 (PMC10970649; doi:10.3390/ijms25063166)
Supplement: Supplementary file 1 [file ijms-25-03166-s001.zip › ijms-2825474-supplementary.pdf]

Supplementary Table S1. Primer sequences, both forward and reverse, used in qRT-PCR. Primers were designed with ncbi Primer-BLAST tool and ordered from Integrated DNA Technologies (IDT, Coralville, IA, USA). The full primer sequences are listed here in 5’-3’ orientation along with their associated target gene.

Target	Primer Sequence
**STAT4-F**	5’-GGTGGGAACTGACCCAAGTA-3’
**STAT4-R**	5’- AGACATGCTAGCGCTCTCTC-3’
**STAT1-F**	5’-CTGTGCGTAGCTGCTCCTTT-3’
**STAT1-R**	5’- CCACTGAGACATCCTGCCAC-3’
**GAPDH-F**	5’-AGGGCTGCTTTTAACTCTGGT-3’
**GAPDH-R**	5’-CCCCACTTGATTTTGGAGGGA-3’

Supplementary Table S2. Antibodies for Western blot listed here with source, species and dilution used. Final dilution for each antibody was determined by trial and error and initial dilutions determined by recommendation from the vendor. Primary antibodies were selected based on Western blot quality. Secondary antibodies were applied according to the species of each primary antibody.

Target	Assay	Species	Source	Cat#	Dilution
STAT4	WB	Mouse	SCBT	Sc-398228	1:100
STAT1	WB	Mouse	SCBT	SC-464	1:200
P-STAT4	WB	Mouse	SCBT	SC-28296	1:100
P-STAT1	WB	Rabbit	CST	91675	1:1000
IRF1	WB	Mouse	SCBT	SC-514544	1:100
Cofilin	WB	Mouse	SCBT	SC-376476	1:100
Cyclophilin B	WB	Mouse	SCBT	SC-130626	1:200
GAPDH	WB	Mouse	SCBT	SC-47724	1:200
Anti-mouse IgG-HRP	WB	Horse	CST	7076S	1:3000
Anti-rabbit IgG-HRP	WB	Goat	CST	7074S	1:3000

CST is Cell Signaling Technology; SCBT is Santa Cruz Biotechnology
